# Subchondral Insufficiency Fracture of the Humeral Head: A Rare Case in a Young Person

**DOI:** 10.7759/cureus.101654

**Published:** 2026-01-16

**Authors:** Hirokazu Murayama, Yasuhiro Mizuki

**Affiliations:** 1 Orthopaedic Surgery, Sasebo Kyosai Hospital, Sasebo, JPN

**Keywords:** hemiarthroplasty, humeral head, osteochondral autograft, shoulder pain, subchondral fracture

## Abstract

Subchondral insufficiency fracture (SIF) of the humeral head is rare compared to SIF of the femoral head, and most of the reported cases have been of elderly women with osteoporosis. We report a rare case of SIF of the shoulder in a man aged 32 years. Following a consultation with a local physician, the patient was referred to our hospital with worsening right shoulder pain and suspected humeral head necrosis based on magnetic resonance imaging (MRI) findings. The patient had no history of steroid or excessive alcohol consumption. The patient had a limited range of motion in the shoulder joint and decreased lumbar bone mineral density. Radiography of the right shoulder revealed collapse and deformation of the humeral head. MRI revealed a depressed fracture of the humeral head, partial thinning, and loss of articular cartilage in the glenoid cavity. He had received intra-articular injections of triamcinolone acetonide, which yielded no improvement; therefore, hemiarthroplasty and osteochondral autografting were performed. SIF was diagnosed based on the pathology of the excised head, which exhibited callus formation and granulation tissue. Two years after surgery, his range of motion and pain improved. This study demonstrates that it is important to consider SIF as a differential diagnosis for shoulder pain, even in young men.

## Introduction

Subchondral insufficiency fracture (SIF) of the humeral head is characterized by the occurrence of fractures in the subchondral area of the joint. Most patients who have humeral SIF are elderly women with osteoporosis [1]. There have been several reports of SIF in the femoral head of young patients [2,3]. By contrast, to the best of our knowledge, cases of SIF in the humeral head of young patients have not been reported. In this report, we described a rare case of SIF in the shoulder of a 32-year-old man. Informed consent for publication of the case was obtained from the patient.

## Case presentation

**Figure 1:**
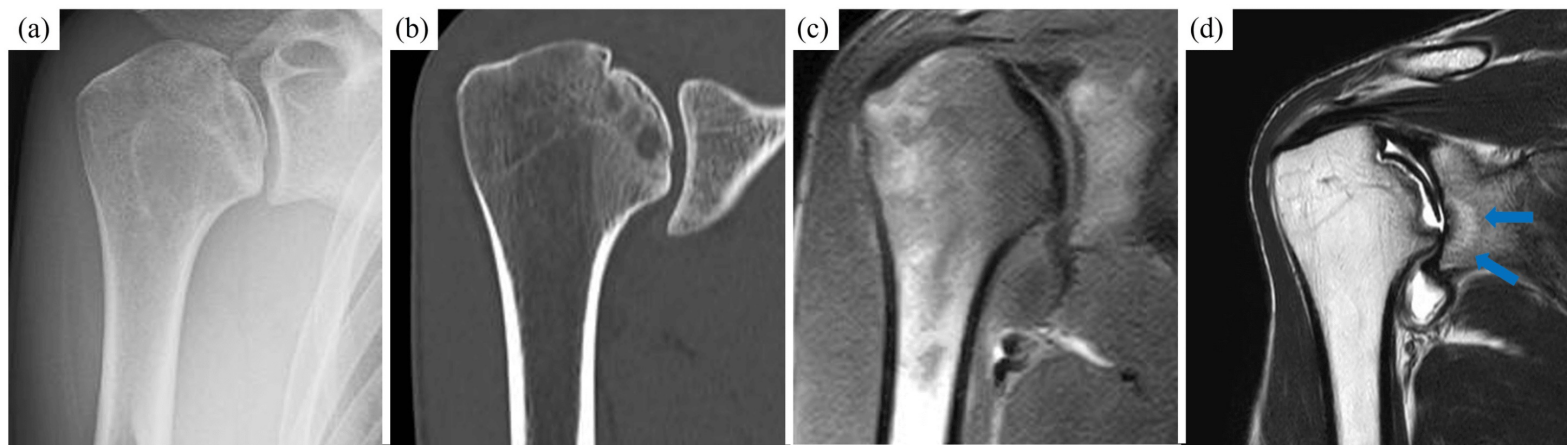
Preoperative images. Preoperative radiograph (a) and CT image (b) revealed collapse and deformation. MRI revealed a depression fracture of the humeral head on the articular surface, bone marrow edema (c), and joint effusion with a high signal area on the side of the glenoid cavity (d, arrows). No obvious low band was observed.

A 32-year-old man visited a local physician because of right shoulder pain and limited range of motion. MRI revealed suspected osteonecrosis of the humeral head, and the patient was referred to our hospital. The patient had no history of trauma, steroid use, or heavy alcohol consumption, and he had not engaged in heavy work or sports activities. The active range of motion values for the right shoulder compared with the left shoulder were as follows: forward flexion, 45° versus 170°; abduction, 45° versus 170°; external rotation, 5° versus 70°; and internal rotation, L1 versus T6. Scores for shoulder function on the University of California, Los Angeles, and Japanese Orthopaedic Association assessments were 10 and 52 points, respectively. Radiography of the right shoulder revealed collapse and deformation of the humeral head. MRI revealed a depressed fracture of the humeral head on the articular surface (Fig. 1). Moreover, partial thinning and loss of articular cartilage were observed in the glenoid cavity. Mild osteoarthritis of the shoulder was also suspected.

The bone mineral density of the lumbar spine was low (0.923 g/cm2). Although electrolyte values were within normal ranges, bone metabolism markers exhibited abnormalities with serum N-terminal telopeptide at 31.7 nmol (normal 9.5-17.7 nmol) bone collagen equivalents per 1 mmol/L creatinine, undercarboxylated osteocalcin at 6.10 ng/mL (normal 0.00-4.49 ng/mL), and procollagen type 1 N-terminal propeptide at 118.3 ng/mL (normal 18.1-74.1 ng/mL). Laboratory tests showed a white blood cell count of 4,830/μL (normal 3500-9000/μL) and a C-reactive protein level of 0.05 mg/dL (0.00-0.14 mg/dL); therefore, active infection was not suspected.

He had received intra-articular injections of triamcinolone acetonide in the right shoulder at the initial consultation and again at three and five weeks after the first visit. However, as his condition did not improve, surgery was performed six months after the initial consultation. Considering the patient’s young age and the relatively preserved glenoid cartilage, hemiarthroplasty was chosen to preserve bone stock and avoid the complications of total shoulder arthroplasty in young patients.

**Figure 2:**
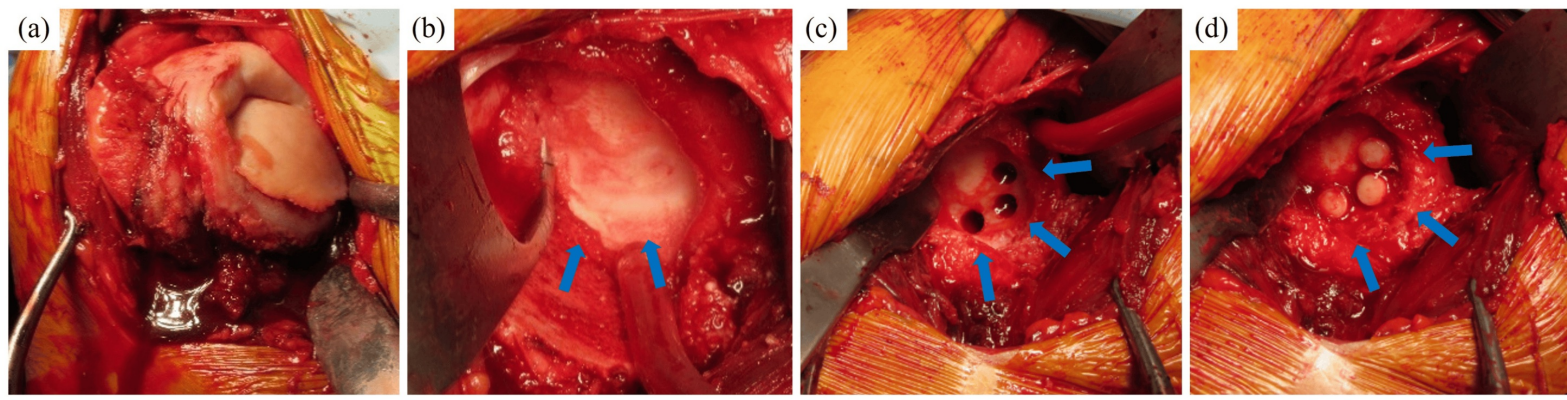
Intraoperative findings. The humeral head was crushed (a). The arrows in (b) indicate that the anterior inferior part of the glenoid cavity was worn and had a step-like appearance. Three bone-cartilage columns were transplanted from the knee (c) into the glenoid cavity (d).

**Figure 3:**
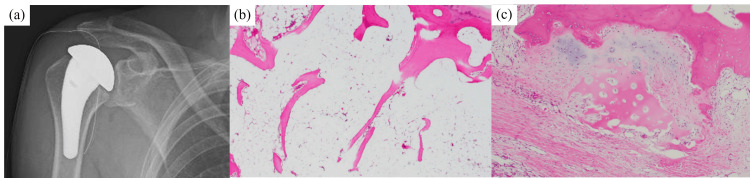
Postoperative image and pathological findings. Plain radiograph after hemiarthroplasty and osteochondral autografting (a).  Pathological findings revealed a decrease in the number of trabeculae (b) along with callus formation and tissue granulation (c). These findings led to a diagnosis of subchondral insufficiency fracture.

With the patient under general anesthesia and in the beach chair position, the deltopectoral approach was used for the right shoulder. The rotator cuff was intact, whereas the subscapularis muscle was partially detached and was later reconstructed. The humeral head was depressed and collapsed, and the cartilaginous surface had become flap-like. In the glenoid fossa, the lower part of the cartilage was lost, exposing the subchondral bone. The gap between the upper part and the remaining part of the cartilage was approximately 2 mm. Three autografts were harvested from the right knee and transplanted into the glenoid cavity (Fig. 2). The surgical procedure was completed with hemiarthroplasty (Fig. 3). The excised humeral head was pathologically evaluated. Although no clear signs of necrosis were observed, a decrease in the number of trabeculae and the formation of callus were observed (Fig. 3). Therefore, a histological diagnosis of SIF was made.

**Figure 4:**
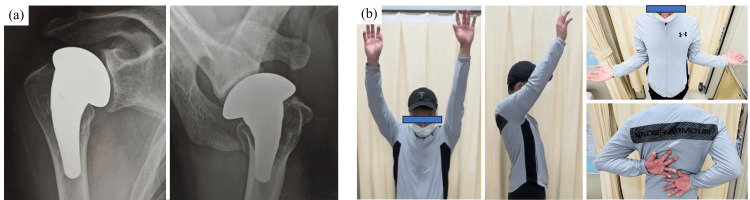
Radiographic findings and shoulder range of motion two years after surgery. Two years after surgery, radiography (a) revealed continued good fixation of the implants, no peri-implant fractures, and fusion of the osteochondral grafts. Active range of motion of the shoulder joint 2 years after surgery (b) was as follows: forward flexion, 120° versus 170°; abduction, 125° versus 170°; external rotation, 40° versus 70°; internal rotation, T10 versus T6.

After surgery, the right upper limb was immobilized with a triangular bandage for four weeks, except for passive range of motion exercises up to 90° of flexion and 30° external rotation and muscle strengthening exercises. From four to six weeks after surgery, assisted active range of motion exercises were performed, and from six weeks after surgery, active range of motion exercises were performed without restriction. He was initiated on oral bisphosphonate therapy by a local physician because of decreased bone mineral density. Two years after surgery, radiographs revealed no loosening of the implants and no peri-implant fractures, and the osteochondral grafts exhibited signs of fusion (Fig. 4). The active range of motion had improved as follows: forward flexion, 120° versus 170°; abduction, 125° versus 170°; external rotation, 40° versus 70°; internal rotation, T10 versus T6 (Fig. 4). The University of California, Los Angeles, shoulder score was 21, and the Japanese Orthopaedic Association score was 76 points, showing improvement compared to before the surgery.

## Discussion

Subchondral insufficiency fracture is a fragility fracture that occurs beneath the articular cartilage. It is most often seen in the hip [4] and more rarely in the shoulder [1, 5]. Previous case studies of SIF in the humeral head have described older women with reduced bone density [6]. One of the important differential diagnoses is osteonecrosis of the head of the humerus [6]. Although not yet well described in the shoulder owing to humeral head collapse in previous case reports [6,7], SIF is well characterized in the hip. Those fractures occur most frequently in elderly women with osteoporosis, and on MRI, the band image is irregular, discontinuous, and convex in the center [5,8]. By contrast, osteonecrosis of the humeral head occurs in men and women aged 20-60 years with a history of steroid or excessive alcohol use, and the MRI band image is smooth, circumferential, and convex in shape. The difference in shape is attributed to the fact that the band image in SIF indicates a fracture line, whereas in osteonecrosis of the head, it indicates a repair reaction [4,8]. However, as seen herein and in previous reports, the head of the shoulder joint had already collapsed when MRI imaging was performed, making it difficult to evaluate the shape of the band [6,7].

The non-weight-bearing nature of the shoulder joint may delay humeral head collapse in SIF. If no abnormalities are found on radiographs, a detailed examination may not be performed without an MRI, delaying SIF diagnosis. In this case, it was difficult to make a differential diagnosis based on imaging findings. However, SIF was suspected because the patient had no history of steroid use or heavy alcohol consumption, a decrease in bone mineral density, and a definitive diagnosis was reached histologically. In this case, the bone metabolic markers indicated increased activity of both bone resorption and bone formation, suggesting a high bone turnover state [9,10]. Although the abnormalities in bone metabolic markers are presumed to be secondary to the fragility fracture, thyroid function and vitamin D deficiency were not assessed; therefore, other potential etiologies of bone fragility cannot be excluded.

Treatment of SIF of the humeral head is not well described due to the rarity of this condition. Hemiarthroplasty and total shoulder arthroplasty are the main treatments for osteoarthritis secondary to collapse of the humeral head, and the degree of pain relief and improvement in the range of motion are similar for these two surgical procedures [11]. However, Schoch et al. [12] reported that the implant survival rates for hemiarthroplasty were 90% at 10 years and 87.4% at 20 years; however, the implant survival rates for total shoulder arthroplasty were 83% at 10 years and 78.8% at 20 years. Thus, hemiarthroplasty was recommended when the articular surface of the glenoid fossa was acceptable. In this case report, partial osteoarthritis of the glenohumeral joint was observed due to partial loss and collapse of the articular cartilage in the glenoid cavity. However, because the patient was young, we attempted to preserve the bone by performing an osteochondral autograft in the glenoid. Autograft transplantation has led to long-term satisfaction with outcomes in young patients after surgery for various osteochondral conditions [13].

## Conclusions

In conclusion, we experienced a rare case of SIF of the humeral head in a young patient. Differentiation from osteonecrosis is essential, and SIF should be considered in patients without risk factors such as steroid or excessive alcohol use. Careful evaluation with bone mineral density and metabolic markers is recommended, as some cases may respond to conservative treatment. This case demonstrates that osteochondral autografting can preserve the glenoid bone and may be a useful approach in young patients.
